# Comparison of Local and Systemic DTPA Treatment Efficacy According to Actinide Physicochemical Properties Following Lung or Wound Contamination in the Rat

**DOI:** 10.3389/fphar.2021.635792

**Published:** 2021-03-26

**Authors:** Nina M. Griffiths, Anne Van der Meeren, Olivier Grémy

**Affiliations:** Laboratoire de RadioToxicologie, CEA, Université de Paris-Saclay, Bruyères le Châtel, France

**Keywords:** actinides, contamination, decorporation, DTPA, lung, wound

## Abstract

**Purpose:** In cases of occupational accidents in nuclear facilities or subsequent to terrorist activities, the most likely routes of internal contamination with alpha-particle emitting actinides, such as plutonium (Pu) and americium (Am), are by inhalation or following wounding. Following contamination, actinide transfer to the circulation and subsequent deposition in skeleton and liver depends primarily on the physicochemical nature of the compound. The treatment remit following internal contamination is to decrease actinide retention and in consequence potential health risks, both at the contamination site and in systemic retention organs as well as to promote elimination. The only approved drug for decorporation of Pu and Am is the metal chelator diethylenetriaminepentaacetic acid (DTPA). However, a limited efficacy of DTPA has been reported following contamination with insoluble actinides, irrespective of the contamination route. The objectives of this work are to evaluate the efficacy of prompt local and/or systemic DTPA treatment regimens following lung or wound contamination by actinides with differing solubility. The conclusions are drawn from retrospective analysis of experimental studies carried out over 10 years.

**Materials and Methods:** Rat lungs or wounds were contaminated either with poorly soluble Mixed OXide (U, Pu O_2_) or more soluble forms of Pu (nitrate or citrate). DTPA treatment was administered promptly after contamination, locally to lungs by insufflation of a powder or inhalation of aerosolized solution or by injection directly into the wound site. Intravenous injections of DTPA were given either once or repeated in combination with the local treatment. Doses ranged from 1 to 30 µmol/kg. Animals were euthanized from day 7–21 and alpha activity levels were measured in urine, lungs, wound, bone and liver for determination of decorporation efficacy.

**Results:** Different experiments confirmed that whatever the route of contamination, most of the activity is retained at the entry site after insoluble MOX contamination as compared with contamination with more soluble forms which results in very low activities reaching the systemic compartment and subsequent retention in bone and liver. Several DTPA treatment regimens were evaluated that had no significant effect on either lung or wound levels compared with untreated animals. In contrast, in all cases systemic retention (skeleton and liver) was reduced and urinary excretion were enhanced irrespective of the contamination route or DTPA treatment regimen.

**Conclusion:** The present study demonstrates that despite limitation of retention in systemic organs, different DTPA protocols were ineffective in removing insoluble actinides deposited in lungs or wound site. For moderately soluble actinides, local or intravenous DTPA treatment reduced activity levels both at contamination and at systemic sites.

## Introduction

Internal contamination with high-energy alpha particle emitters such as the actinides plutonium (Pu) or americium (Am), presents a challenge to the design and application of radiation medical countermeasures. In contrast to external gamma radiation exposure, internal contamination following either inhalation or wounding results in deposition of radioactive elements at the primary site of contamination, i.e. lungs or injury site, as well as in systemic target tissues that retain these elements. The primary objective of countermeasures is therefore to reduce these radioactivity levels. Even if stringent radiation protection measures are taken internal contamination with Pu/Am remains a potential hazard for workers involved in various stages of the nuclear fuel cycle such as preparation and reprocessing of used fuel, treatment/conditioning of waste as well as reactor decommissioning. Furthermore, in the event of an attack with a Radiological Dispersal Device or “Dirty Bomb” dissemination of actinides would be a potential health hazard for the general public and first responders resulting in serious radioactive contamination. Contamination by inhalation of aerosols may be associated with exposure of higher numbers of people as compared with contamination after wounding.

Cases of actinide contamination by inhalation have been reported since the initial use of compounds for either military devices or nuclear fuels (Carbaugh and La Bone, 2003; Okladnikova et al., 2005; Grappin et al., 2007). Similarly, accidental entry of actinides following wounding has also been reported (Ilyin, 2001; Falk et al., 2006; James et al., 2007; Sugarman et al., 2018; Klumpp et al., 2020). Indeed, case reports of incidents/accidents, biological assay data and tissues have been collated by registries in several countries (Loffredo et al., 2017; Kathren and Tolmachev, 2019) that provide a valuable source of information.

Inhalation and subsequent deposition of actinides in deep lung compartments results partly in transfer to the bloodstream. The size of this transferable fraction is dependent on a number of variables but in particular, the physicochemical nature of the actinide compounds. In the case of skin contamination, under normal healthy conditions, these compounds do not cross the epidermal skin barrier easily. However, this may be significantly increased after wounding associated with concomitant physical insults that result in loss of skin barrier function. This may be associated with strongly acidic actinide solutions that result in burns as was the case at Rocky Flats in 1965 and Hanford in 1976 (Lagerquist et al., 1967; McMurray, 1983). Such cases of actinide and acid are a dual insult to the skin barrier. Whatever the primary site of contamination (lungs or wound) and subsequent entry into the general circulation, the main sites of secondary long-term retention for Pu and Am are the skeleton and liver (International Commission on Radiological Protection (ICRP), 1986). A small fraction of circulating actinides will be excreted predominantly in urine with minor levels in faeces. More soluble compounds will be transferred more rapidly and in a larger proportion, whether after inhalation or wounding (International Commission on Radiological Protection (ICRP), 1986; National Council on Radiological Protection and Measurements (NCRP), 2006).

The question of the fate of tissue-retained actinides, even initially soluble ones, has been studied particularly with regard to deposition and retention in lungs and eventual effective dose (International Commission on Radiological Protection(ICRP), 1994a). Retention compartments in the lungs can be macrophages, interstitial connective tissue as well as scar tissue (Hahn et al., 2004; Van der Meeren et al., 2012; Van der Meeren et al., 2014; Lamart et al., 2017; Birchall et al., 2019). Regardless of contaminant solubility (from soluble to insoluble forms such as oxides and metals), retained activity may be considered as a “reservoir”. Actinides in this “reservoir” may be very slowly dissolved, absorbed into the blood and afterwards transferred to systemic organs so contributing to the systemic long-term effective radiation dose. Countermeasures are also required to address these “reservoir” problems that contribute to the long-term effects of an inhomogeneous chronic irradiation. Following inhalation of actinides pneumonitis, lung fibrosis and tumour development have been observed in a number of species (rat, mouse and dog) (Dudoignon et al., 2003; Muggenburg et al., 2008; Griffiths et al., 2010), as well as in man (Hahn et al., 2004; Newman et al., 2005; Sychugov et al., 2020). The pathological consequences after wounding have been less well-studied. However, a clinical report by Lushbaugh and Langham (1962) observed cutaneous necrosis and fibrosis around the highly active Pu deposit.

As a countermeasure and regardless of the Pu/Am compound and the route of intake, the only approved treatment for decorporation (removal from the body) of Pu/Am in man remains diethylenetriaminepentaacetic acid (DTPA) as the calcium or zinc salt (Food and Drug Administration, 2003; Grappin and Bérard, 2008). DTPA is a metal chelator that has a high affinity in particular for Pu and Am and first published data on the efficacy of this compound to remove (decorporate) these elements appeared in the 1960s (Norwood, 1960). DTPA is a highly charged acidic compound that has a short plasma half-life, is poorly absorbed and is rapidly eliminated by glomerular filtration. It is considered to circulate mainly in the extracellular fluids where it is able to chelate actinides. In general and as recommended the DTPA solution is administered as soon as possible for the most part by intravenous injection or infusion. Inhalation of the aerosolized DTPA solution is only recommended in cases of contamination by inhalation (Food and Drug Administration, 2003; ANSM, 2011). Experimental DTPA formulations have also been tested in rodents such as liposomes for cell entry enhancement (Grémy et al., 2018) or a dry powder for better delivery into deep lung (Grémy et al., 2010; Grémy et al., 2012).

For local treatment of contaminated wounds, DTPA solution is used for external decontamination as well as for irrigation of the wound site. In addition, intravenous DTPA administration is used concomitantly where excision of contaminated wound is necessary (National Council on Radiological Protection and Measurements. (NCRP), 2009). The beneficial effects of local injection of DTPA to a contaminated wound site have been little studied to date although animal studies have demonstrated this route to be effective (Volf, 1974; Harrison and David, 1979; Stradling et al., 1993; Griffiths et al., 2014).

Many studies have been carried out on the effect of different regimens of DTPA treatment following contamination by inhalation or wounding with single actinides under different chemical forms (oxides, nitrate) but only few studies to date have been carried out following contamination by Mixed U, Pu OXide (MOX) that is used in the nuclear fuel cycle.

This paper presents an overview of *in vivo* data obtained in rat for actinide decorporation by different DTPA regimens of varying physicochemical forms. The principal objective was to compare local and systemic DTPA treatment regimens according to actinide physicochemical properties following lung or wound contamination. As far as possible the same treatment protocols (local, systemic or combined) are compared for each actinide compound in either the lung or wound model of contamination. Efficacy was determined from the key parameters of DTPA-induced increase in urinary actinide excretion together with evidence for reduction in actinide retention at the primary site of contamination, i.e., lung or wound site, and in key secondary retention organs, namely bone and liver.

## Materials and Methods

### Chemicals

Marketed DTPA solution as the calcium trisodium salt [Na_3_(Ca-DTPA)] was obtained from the Pharmacie Centrale des Armées (PCA; Orléans, France). DTPA dry powder (75% DTPA) was formulated as previously described in detail elsewhere (Gervelas et al., 2007). This powder has good aerosolization properties due to a median geometric diameter of 4.5 µm and a “crumpled paper” morphology (Gervelas et al., 2007) allowing access to deep alveolar compartments.

### Preparation of Actinide Contaminants

Plutonium used for experiments was obtained from two laboratory stock solutions of Pu kept in 2 M HNO_3_, acquired from the French Alternative Energies and Atomic Energy Commission (CEA).

#### Pu Nitrate Solution for Contamination

After evaporation of an aliquot of the first stock Pu solution (86.1% ^238^Pu, 12.5% ^239^Pu), Pu was dissolved in distilled water to have a working Pu nitrate solution with low nitrate.

#### Pu Citrate Solution for Contamination

After Pu purification by anion exchange chromatography of an aliquot of the second stock Pu solution and evaporation (99.5% ^238^Pu, 0.5% ^239^Pu), Pu was dissolved in diluted citrate so that final Pu:Na-citrate ratio is 1:10,000.

#### MOX Suspension for Contamination

Mixed U, Pu OXide (MOX) powder from the rectification step was produced by the MIcronised MASter Blend (MIMAS) procedure at the MELOX installation (Marcoule, France) containing 81% U and 7.1% Pu by mass. At the time of experimentation, the specific activity of the MOX powder was 123.4 kBq/mg and contained ^241^Am due to aging from ^241^Pu decay. In terms of mass of each isotope and element (Pu + Am) the composition was 1.7% ^238^Pu, 88.1% ^239+240+241^Pu, and 4.1% ^241^Am. As a percentage of total Pu plus Am alpha activity ^238^Pu represented 55%, ^239+240+241^Pu 18% and 27% ^241^Am. For aerosol generation, a suspension of MOX powder in ethanol 100% was diluted using distilled water. For the wound deposit an aliquot of suspension (in ethanol 100%) was used which was diluted in saline (0.9% NaCl) before use. Care was taken to maintain the particles in suspension.

### Animals

All the data were obtained from *in vivo* experiments spanning over more than 10 years. In some cases methods have changed, analytical procedures have been upgraded and regulations have been updated. However basic experimental approaches have not changed and are reported as such.

Male Sprague-Dawley rats weighing between 200–450 g were obtained from Charles River, L’Arbresle Cedex France. Animals were maintained at constant temperature (20–24°C), humidity and lighting (12 h light -12 h dark) and fed standard rat chow and water ad libitum. Cages contained tunnels, paper and wood for gnawing to provide enrichment of the environment. General health status and weight was assessed regularly throughout the duration of the experimental period.

For euthanasia animals received buprenorphine (0.02 mg/kg, s.c.; Buprecare, Axience, France), and were injected with a lethal dose sodium pentobarbital (400 mg/kg, i.p. Exagon, Axience, France) followed by exsanguination from the dorsal aorta or following intra-cardiac puncture.

All experiments were carried out in an accredited facility according to French regulations for animal experimentation under the European directives (2001-246 June 6, 2001 and 2010/63/EU, September 22, 2010). Experiments were approved by the local institutional animal ethics committee and the French Ministry of National Education, Higher Education and Research.

### Contamination Procedures

#### Pulmonary Contamination of Rats by Pu Citrate or Pu Nitrate

Under light gaseous anesthesia (2.5% isoflurane; Aerrane, Baxter, France), rats were contaminated by intra-tracheal instillation of a 200–250 µl volume containing Pu citrate or Pu nitrate solution.

#### Pulmonary Contamination of Rats by MOX

Conscious rats and restrained in cardboard tubes, were nose-only exposed to a MOX aerosol generated from an aqueous suspension using a compressed air device, as described previously by Andre and colleagues (André et al., 1989). The aerosol had an activity median erodynamic diameter (AMAD) of 4.2 µm and a geometric standard deviation of 2.7.

#### Wound Contamination of Rats by Pu Nitrate or MOX

For contamination after wounding, animals were anesthetized using sodium pentobarbitone (40 mg/kg, i.p) The left hind leg was clipped and an incision (0.5 cm long, 0.4–0.7 cm deep) using a scalpel (N° 11) was made in the interior aspect of the hind limb. This technique has been previously described in detail (Beitz et al., 2004; Griffiths et al., 2012). Pu nitrate solution (50 µl containing 5–10 kBq) or a suspension of MOX (50 µl containing 20–30 kBq in aqueous solution) was then introduced using a micropipette (Gilson 0–100 µl) and a sterilized cone. The wound was then closed and sutured using resorbable thread and the animals allowed to recover from anesthesia. All animals received anti-inflammatory treatment after contamination using Tolfedine (Vetoquinol, Lure, France; 4 mg/kg, s.c.) or Meloxicam (Metacam, Boehringer Ingelheim, Lyon, France; 1 mg/kg, s.c)

### DTPA Treatment Regimens

(a)  For pulmonary-contaminated rats:

Two hours following lung contamination with Pu nitrate, animals received either intravenous injection of DTPA solution (“DTPA i.v.” group; 30 µmol/kg) or pulmonary insufflation of DTPA powder (“DTPA local” group) using a special device (model DP-4, Penn-Century^TM^). Both insufflation and injection into the lateral tail vein of DTPA were carried out under light gaseous anaesthesia (Isoflurane 2.5%). According to a previous study on this DTPA dry powder, only about 26% may reach deep lung compartments (Gervelas et al., 2007). Thus, insufflation at 20 µmol/kg tested in the present study was estimated to result in a deep lung deposit of approximately 5 µmol/kg of DTPA.

One hour following lung contamination with Pu citrate, animals were either injected with DTPA solution (“DTPA i.v.” group; 15 µmol/kg) or nose-only exposed to an aerosol of DTPA solution (“DTPA local” group) diluted in NaCl 0.9% (deep lung deposit of 1.1 µmol/kg), by using an inhalation chamber associated with an Aeroneb® lab micropump nebulizer (technology of a microperforated vibrating membrane; Tem Sega, Pessac, France). The exposure apparatus and DTPA dose determination with ^111^In have been reported in detail elsewhere (Miccoli et al., 2019).

Two hours following lung contamination with MOX, animals received pulmonary insufflation of DTPA powder (“DTPA local” group; deep lung deposit of about 5 µmol/kg), either alone or starting at day 1 by repeated DTPA intravenous injections (30 µmol/kg) given twice a week from day 1 to day 20 (“DTPA local + i.v”. group).

(b)  For wound-contaminated rats:

In an accidental situation DTPA treatment of wounds involves washing/flushing for decontamination purposes in addition to systemic DTPA by i.v. injection. In this study the “intra wound-site” was chosen as the way to administer DTPA locally in a controlled manner. This was to simplify the procedure *in vivo* as well as radiation protection issues. Previous studies have shown this to be effective in animals but no data seem to be available for man (Taylor and Sowby, 1962; Volf 1974; Harrison and David, 1979; Stradling et al., 1993; Griffiths et al., 2014).

At 2 h following wound contamination with Pu nitrate, animals received either a single local injection (“DTPA local” group) into the wound site using a Hamilton syringe (0–100 µl; 30G needle; 30 µmol/kg) or a systemic injection of DTPA (“DTPA i.v.” group; 30 µmol/kg).

Similarly at 2 h following wound contamination with MOX animals received a single local DTPA injection in the wound site (“DTPA local” group; 30 µmol/kg), either alone or followed one day later by i.v. DTPA administration (30 µmol/kg). Further DTPA i.v. injections were given twice a week up to day 20 (“DTPA local + i.v.” group).

In all cases where DTPA was injected at the wound site which may cause local pain, the DTPA solution contained the local anesthetic lidocaine at a final concentration of 0.5% (Xylovet, Ceva Santé Animal, Libourne, France).

### Excreta Collection, Tissue Sampling and Activity Measurements

Following contamination animals were housed in metabolism cages for collection of urine and/or faecal samples. At euthanasia at 7 or 21 days depending on studies, the liver, lungs, femurs were removed for radioactivity analyses. In some pulmonary-contaminated rats, a bronchoalveolar lavage was carried out for measurement of macrophage-associated activity as previously described (Van der Meeren et al., 2012).

For measurement of activity, tissue samples were dry-ashed (500–600°C depending on sample) and wet ashed in HNO_3_ (2 M) and H_2_O_2_ (30%) until a clear solution was obtained. Urine samples were evaporated to dryness and then mineralized as for tissues. The dry residues were taken up into HNO_3_ (2–4 ml, 2 M) and an aliquot used for determination of total alpha activity by liquid scintillation counting (Packard Tri-Carb 2500). For tissues containing low levels of radioactivity and for measurement of Pu and Am, samples were analyzed by alpha spectrometry following separation of the two elements by anion exchange (Tru-Spek columns, Eichrom, Rennes, France).

For pulmonary contamination by intratracheal instillation of Pu nitrate/citrate, the Initial Lung Deposit was determined as the activity administered into airways after subtracting it from the activity recovered in in faeces during the first two days after contamination as described previously (Grémy et al., 2012). For pulmonary contamination by MOX inhalation, determination of Initial Lung Deposit was determined by gamma-ray spectrometry of thorax using a NaI detector as MOX contains Am with a gamma signal at 59 keV. This measure occurred only seven days after MOX inhalation so that larger particles initially deposited in the upper airways were eliminated by mucociliary clearance. Initial wound radioactivity (T = 0) was determined by total limb counting using a NaI detector with the anesthetized rat positioned so that the contaminated wound site was within the detector area. Corrections were made for absorption in air and tissue using a tissue-equivalent phantom. The height of the leg and distance from the detector surface was measured in each case. For Pu contaminated wounds using Pu nitrate solution initial activity was determined from the known administered activity minus activity collected on a cleaning swab of the area.

### Data Presentation and Analyses

Data are expressed as mean percentage of the Initial Wound Deposit (wounds) or the Initial Lung Deposit (inhalation) in activity ± SD for 3–6 animals.

For comparative purposes reduced tissue activity are expressed as the percentage change as compared with contaminated untreated animals. This referred to as percentage inhibition (tissues). Significant differences between the different exposure groups were compared using the unpaired Student’s *t* test.

## Results and Discussion

### Single Systemic or Local DTPA Treatment Following Contamination of Lungs or Wound With Pu Nitrate or Pu Citrate

#### Contamination of Lungs

**FIGURE 1:**
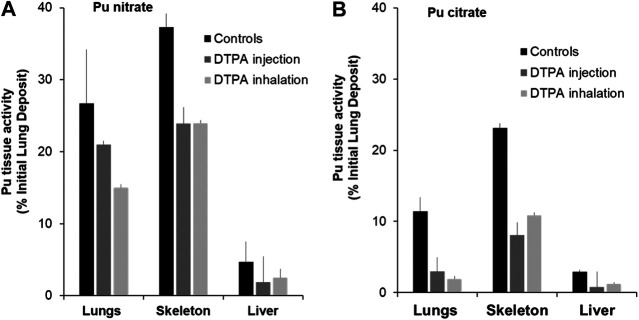
Effect of single systemic or local DTPA administration on tissue activity levels following lung contamination with Pu nitrate **(A)** or Pu citrate **(B)**. Animals were contaminated by intra-tracheal instillation of either Pu nitrate (**A**: 5.5 kBq) or Pu citrate (**B**: 2.8 kBq) and were euthanized at eight or seven days respectively after contamination. After Pu nitrate contamination **(A)**, treatments at 2 h were either intravenous injection of DTPA solution (30 µmol/kg) or insufflation of DTPA powder (approximatively 5 µmol/kg). After Pu citrate contamination **(B)**, treatments at 1 h were either intravenous injection of DTPA solution (15 µmol/kg) or inhalation of nebulized DTPA (1.1 µmol/kg). Data are expressed as a percentage of the initial lung deposit and are the means of three–six animals.

Figure 1 shows data obtained from studies to investigate early decorporation efficacy of systemic or locally administered DTPA after pulmonary contamination with Pu nitrate (Figure 1A) or Pu citrate (Figure 1B). Firstly, it should be noted that in both cases a significant amount of activity is retained in the lungs even with the more soluble citrate form. Secondly, as expected in control untreated animals, bone retained higher activities than liver. In order to compare the data concerning the efficacy of the two routes of DTPA administration, i.e., systemic (i.v. of DTPA solution) and pulmonary (insufflation of DTPA dry powder or inhalation of aerosolized DTPA solution), percentage inhibitions of plutonium tissue retention were calculated as compared to untreated animals. Both DTPA administration routes reduce tissue Pu activity. It appears when given rapidly as a single dose within one or 2 h the local pulmonary route is at least as effective as the intravenous route to reduce lung Pu levels (44% inhibition compared with 21% for Pu nitrate (Table 1) and 83% compared with 74% for Pu citrate).

**TABLE 1:** Reduction of Pu tissue activity levels following single local or systemic DTPA treatment.

Contaminant	DTPA treatment	Wound			Lungs		
		Leg	Skeleton	Liver	Lungs	Skeleton	Liver
Pu nitrate	i.v.	36 ± 15	56 ± 10	80 ± 3	21 ± 7	36 ± 6	60 ± 7
	Local	48 ± 8	49 ± 6	76 ± 3	44 ± 10	36 ± 10	46 ± 25

Animals received Pu nitrate by either intratracheal instillation or following wounding and were euthanized at seven days after contamination. After contamination, treatments at 2 h were either intravenous injection of DTPA solution (“i.v.”; 30 µmol/kg) or local administration, either by insufflation of DTPA powder (“Local”; approximately 5 µmol/kg) or injection into the wound site (“Local”; 30 µmol/kg). Data are from five to six animals and are expressed as a percentage reduction as compared with contaminated, untreated animals.

In addition it should be noted that inhaled DTPA doses (about 5 µmol/kg for DTPA dry powder insufflation and 1.1 µmol/kg for aerosolized DTPA inhalation) were less than i.v.-administered doses (30 or 15 µmol/kg). Therefore, the bioavailability of DTPA in lungs is better when treatment is local rather than systemic even when the latter is administered at higher doses. Nevertheless, given the reduction in lung activity there is a limited transfer of DTPA given intravenously from blood to lungs that clearly can remove available Pu from lungs.

Pulmonary-administered DTPA reduces Pu burden not only in lungs but also in the liver and bones. This undoubtedly results mainly from chelation of transferable Pu that comprises a large fraction still present in the lungs at early times. The Pu-DTPA complexes formed locally in the lungs will be absorbed into the circulation and then excreted in urine, so preventing systemic tissue deposits. A further explication is that free DTPA can cross the alveolar-capillary barrier to chelate circulating or loosely tissue-bound Pu that will also contribute to a reduction in systemic tissue retention. Indeed it appears that DTPA administered to the lungs, even at a lower dose than the i.v. administration is equally effective. There are no significant differences in bone or liver Pu levels whatever the route of administration (Figures 1A,B).

Regardless of the treatment route, it is noteworthy that early chelation efficacy appears lower after pulmonary contamination with Pu nitrate than after Pu citrate as it is a chemical form less soluble, and hence less accessible for chelation.

#### Contamination of Wounds

**FIGURE 2:**
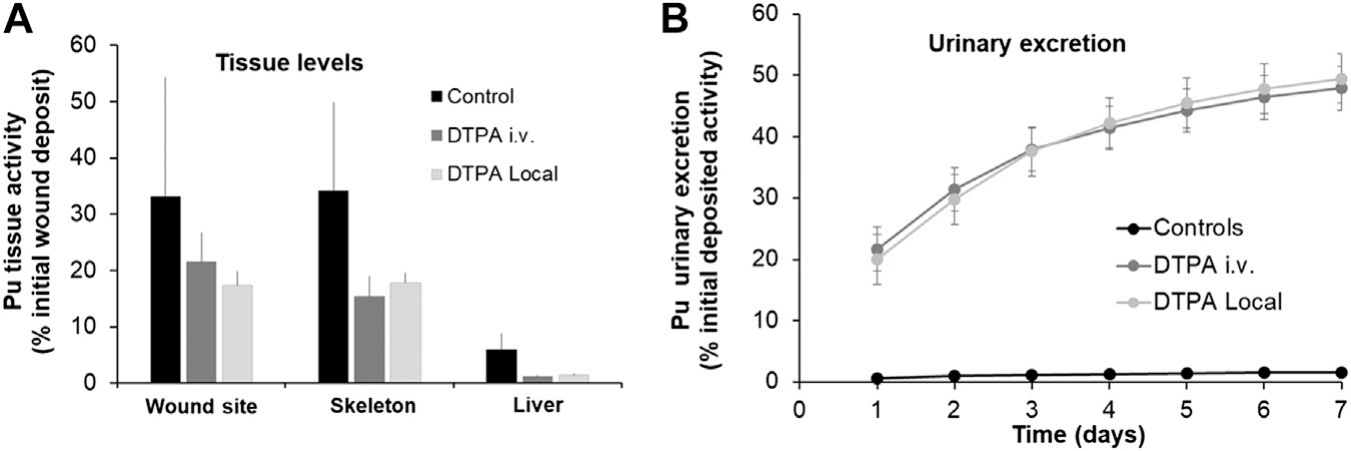
Effect of single systemic or local DTPA administration on tissue **(A)** and urine **(B)** activity levels following wound contamination with Pu nitrate. Animals were contaminated following an incisional wound with Pu nitrate (6 kBq) and were euthanized at seven days after contamination. Urines were collected over seven days. DTPA treatment (30 µmol/kg) by intravenous (“DTPA i.v.”) or local injection (“DTPA Local”) was given at 2 h. Data are expressed as a percentage of the initial wound deposit and are the means ± S.D. of 5–6 animals.

A similar approach was used to study the effects of prompt systemic or local DTPA administration following Pu nitrate contamination of wounds. Similar to the data shown above for lungs, both treatment protocols reduced wound site activity, bone and liver retention (Figure 2A). In agreement with a significant reduction in actinide retention, urinary excretion was enhanced following a single dose of DTPA either given locally or by intravenous injection at similar dosage (Figure 2B). The figure demonstrates a similar efficacy of either systemic or local treatment with regard to reduction in wound and systemic tissue retention (in skeleton: 56 and 49% respectively and in liver: 80 and 76% respectively; Table 1) and increase in urinary excretion (Figures 2A,B).

Similar to the experiments reported above for Pu lung contamination, DTPA was administered early (2 h) after wound contamination when a substantial part of the transferable fraction of Pu nitrate is still present at the wound site. The decrease in systemic Pu observed after DTPA injection into the wound site probably results mainly from a significant chelation of accessible Pu prior to transfer to the circulation. Free DTPA could also be transferred to chelate circulating Pu. This would contribute additionally to the reduction of bone and liver retention.

With regard to DTPA i.v. injection chelation will take place mainly in the systemic compartment. However, it is possible that circulating DTPA gains access to the wound site, given the type of incisional wound and will chelate accessible transferable Pu. Nevertheless, there are no differences in tissue reduction whatever the route of DTPA administration (Figure 2A; Table 1).

The data obtained with moderately soluble Pu nitrate indicate that a single local lung or wound administration of DTPA is effective in reducing the levels of activity at the primary site of contamination (lung or wound). Table 1 shows the data obtained for these two comparative studies. In order to compare the efficacy of the treatment routes the data are shown as the percentage reduction as compared to the untreated animals. Therefore for Pu nitrate a single dose either intravenous or to the primary site of contamination will reduce tissue retention and consequently committed effective radiation dose.

### Different Actinide Forms and Urinary Excretion Profiles

Incidents of contaminated personnel however may not just involved well-characterized nitrate or citrate forms of a single pure actinide. Given the different stages of the nuclear fuel cycle and treatment of used fuel the probability of exposure to far less soluble compounds, such as oxide forms, is greater. This study was carried out using the poorly soluble nuclear mixed oxide compound MOX, and the results compared to that of Pu nitrate contamination.

**FIGURE 3:**
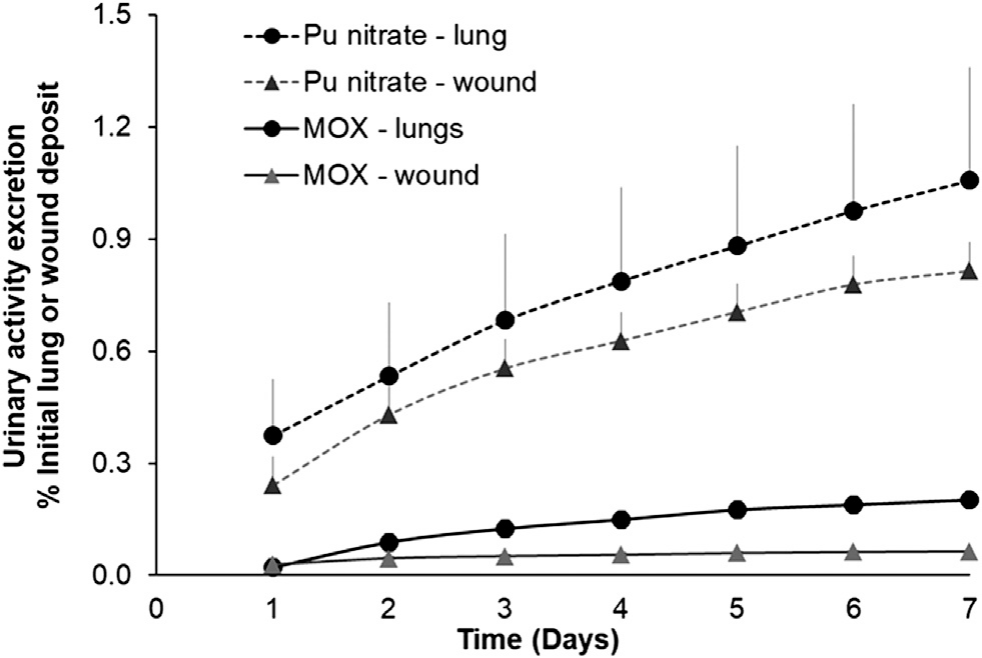
Urinary excretion profiles following lung or wound contamination with MOX or Pu nitrate. Animals were contaminated with MOX by nose-only inhalation (12 kBq) or by a deposit into an incisional wound (13 kBq) as already described. For Pu nitrate pulmonary contamination was by intratracheal instillation (5.5 kBq) or a wound deposit (6 kBq). Data are expressed as a percentage of the initial lung or wound deposit and are the means ± S.D. for three to six animals.

As an example of differing biokinetic profiles of these actinide forms Figure 3 shows cumulative urinary actinide excretion following either lung or wound contamination with MOX or Pu nitrate. The data clearly show differences in urinary excretion between the two physicochemical forms.

Following contamination by inhalation or wounding, the cumulative excretion of Pu nitrate over the seven-day period represented some 1 and 0.7% respectively of the total initial deposit. In contrast, following contamination with MOX cumulative 7-days urinary excretion was some five to ten-fold less than following Pu nitrate (0.2% lung, 0.06% wound). This low excretion is in accordance with the less soluble nature of MOX considered as a type “S” form of inhaled compound (International Commission on Radiological Protection (ICRP), 1994b). Similarly given the particulate nature of MOX it may be considered in the moderate to strong retention category in the NCRP model (2006). This is also in agreement with data obtained for insoluble oxides of Pu after either inhalation (Stather et al., 1982; Grémy et al., 2012) or wounding (Bistline et al., 1974).

For contamination of lungs (12 kBq) or wounds (13 kBq) with the same MOX compound, it seems there is a greater transfer from lungs to the systemic compartment under these experimental conditions as indicated by the higher cumulative urinary excretion (Figure 3). Two factors may contribute to this higher transfer from lungs 1) a significantly greater surface area (around 0.3 m^2^ for rat lung; Fröhlich et al., 2016) for transfer in lungs compared with a very small area in the wound (roughly 0.05 cm^2^) 2) higher blood perfusion leading to higher absorption into the circulation. Nevertheless, this fraction of activity solubilized, absorbed into the blood, then partially excreted is a very small fraction of the total activity administered (<0.2%) since MOX is very predominantly comprised of insoluble particles which are likely to remain at the primary site (lungs, wound) of contamination.

### Prompt Local DTPA Treatment Alone or in Combination With Repeated Systemic DTPA Treatments Following Contamination of Lungs or Wound With MOX

A further study was dedicated to using a local approach followed or not by repeated systemic DTPA injections in order to test the efficacy following contamination of lungs or wound with the poorly soluble MOX.

#### Urinary Activity Excretion

**FIGURE 4:**
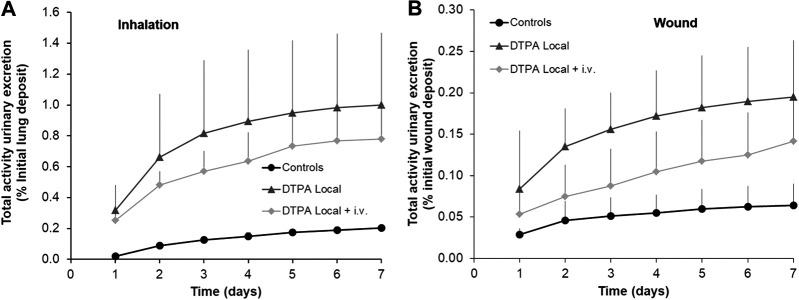
Effect of local DTPA or local DTPA followed by DTPA systemic administrations on cumulative urinary activity excretion following lung **(A)** or wound contamination **(B)** with MOX. Animals were contaminated with MOX either by inhalation (12 kBq) or deposited in a wound (13 kBq) as described. Local administration to lungs (DTPA powder about 5 µmol/kg) or wound (DTPA solution 30 µmol/kg) was started at 2 h (“DTPA Local”). For combined local and systemic DTPA treatment (“DTPA Local + i.v.”), animals received the local dose at 2 h then two intravenous DTPA per week from day one to day 20 (30 µmol/kg). Data are expressed as a percentage of the initial lung or wound deposit and are the means ± S.D. of 3–6 animals.

A single local DTPA treatment administered at 2 h either to lungs (Figure 4A) or to the wound site (Figure 4B) significantly increased the cumulative 7-days urinary excretion of total alpha activity by some 5 and 3 fold respectively. These data are in agreement with other reports following inhalation of Pu oxide or Pu nitrate or simulated wound contamination both in experimental animal studies (Bistline et al., 1974; Guilmette and Muggenburg, 1993; Grémy et al., 2012; Griffiths et al., 2014) and in man (McInroy et al., 1995; Grappin et al., 2007). It can also be observed that cumulative urinary excretion of activity increased exponentially for the first three days and then levels off. This is presumably related in part to MOX low solubility as well as quick elimination of DTPA from the primary site of contamination.

In order to target the slowly dissolving fraction of MOX from the contamination site, repeated i.v. DTPA administration was started at one day after the initial local treatment. However, no further increases were observed in excretion with the combined treatment as compared to the single local treatment as indicated by similar values for cumulative excretion (approximatively 5 fold in inhalation study; approximatively 3 fold in wound study) (Figures 4A,B). These data clearly show the importance and benefit of prompt administration of a single local dose of DTPA. These findings are in agreement with a previous study following inhalation of PuO_2_ followed by local lung DTPA treatment (Grémy et al., 2012). The present work along with this previous study indicates a good bioavailability of DTPA at the primary site of contamination both in the case of early DTPA powder insufflation (in lungs) or early DTPA solution local injection (at the wound site), and hence an expected significant chelation of the transferable fraction of actinides.

In the MOX powder at the time of contamination, Am represents around 27% of total Pu plus Am alpha activity as compared with 13.3% at the time of acquisition (2002). This is due to aging of the supplied powder as a result of the decay of ^241^Pu initially present in the compound. One day after MOX inhalation an enrichment of Am was observed following local DTPA (44 ± 5%) and combined local and i.v. DTPA administration (45 ± 3%). This is in agreement with a previous study where urinary Am levels were increased following inhalation of aged PuO_2_ containing Am (Grémy et al., 2010). Similarly, after contamination by wounding urinary Am was enriched to 67 ± 18% (local DTPA) and 63 ± 8% (local + i.v. DTPA) in the treated groups. There were no differences between the treatment groups.

In lungs or at wound sites locally administered DTPA may compete with same endogenous ligands to form Pu or Am complexes such as citrate or transferrin. Transferrin is accepted to be the main protein ligand for circulating Pu but has less affinity for Am (Ansoborlo et al., 2007). Transferrin is found in lungs to be greater than plasma levels (Bell et al., 1981) due to plasma leakage and local secretion by alveolar type-1 cells (Chen et al., 2006). Transferrin is also an acute phase protein that would be expected to be present rapidly in significant quantities at the incisional wound site (Helson et al., 1983). Consequently, the increased proportion of urinary Am following DTPA treatment may be explained by better chelation availability for Am than for Pu at the primary sites of contamination as well as in the systemic compartment, given the difference between the stability constants of transferrin for the solubilized form of Pu/Am (Ansoborlo et al., 2007).

These observations demonstrate the efficacy of DTPA treatment and are in agreement with other findings both in man and in animals (Newton et al., 1983; Grappin et al., 2007; Sérandour et al., 2007; Grémy et al., 2010). The increased urinary excretion of activity may be explained by chelation of actinides in systemic compartments and/or directly at the primary site of contamination (lungs, wound) followed by transfer to the circulation of the actinide-DTPA complexes and subsequent urinary excretion. At these early times, solubilized Pu/Am available for chelation is still largely within the extracellular space, at the primary site of contamination (lung–epithelial lining fluids- or wound–blood, extracellular matrix) and/or in the circulation within blood/interstitial fluids and/or loosely bound to systemic tissue surfaces. Intracellular chelation would be a possibility but at these early times this would be expected to be negligible as compared to extracellular chelation.

#### Tissue Activity Levels

**FIGURE 5:**
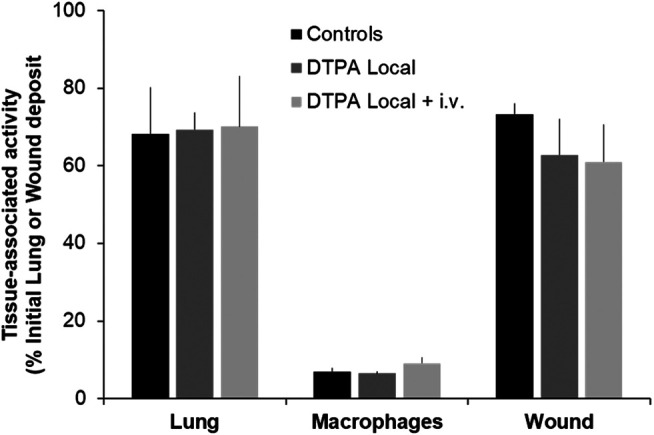
Effect of local DTPA or local DTPA followed by DTPA systemic administrations on activity levels at the primary site of contamination. Animals were contaminated by MOX inhalation (12 kBq) or deposition in a wound (13 kBq) as described, and were euthanized 21 days later. Local administration to lungs (DTPA powder about 5 µmol/kg) or wound (DTPA solution 30 µmol/kg) was started at 2 h (“DTPA Local”). For combined local and systemic DTPA treatment (“DTPA Local + i.v.”) animals received the local dose at 2 h then two intravenous DTPA per week from day one to day 20 (30 µmol/kg). Alveolar macrophages were prepared as described and for data normalization for comparative purposes of DTPA efficacy are expressed as activity per 10^6^ macrophages and as a function of the initial lung deposit. Data are the means ± S.D. of 3–6 animals.

At the end of the 21-days study period, animals were euthanized and tissue levels of total alpha activity measured. It is interesting to note that for a similar activity local deposit (12 kBq lungs and 13 kBq wound) a similar amount of activity remains at 21 days in either case (around 70%) in untreated animals. No significant differences between controls and DTPA-treated animals were seen at the primary site of contamination (lungs or wound) in terms of activity as measured by external counting (Figure 5). Activity levels were also measured in macrophages obtained from bronchoalveolar lavage but as for whole lung, no reduction in macrophage-associated activity was observed whatever the treatment schedule. The absence of a measurable effect on lung or wound activity after treatment is undoubtedly due to the inability of DTPA to dissolve, and hence mobilize, oxides (Grémy et al., 2010) such as MOX that is composed primarily of insoluble oxide particles. As previously shown following inhalation of aged PuO_2_, early insufflation of DTPA powder significantly removed the dissolved fraction within the epithelial lining fluid leading to reduced retention of Pu and Am in bone and liver (Grémy et al., 2010). However this represents an extremely small fraction of the total lung burden (0.1%). Even though local DTPA treatment reduced activity in this fraction changes were undetected by measurement of total lung activity.

**FIGURE 6:**
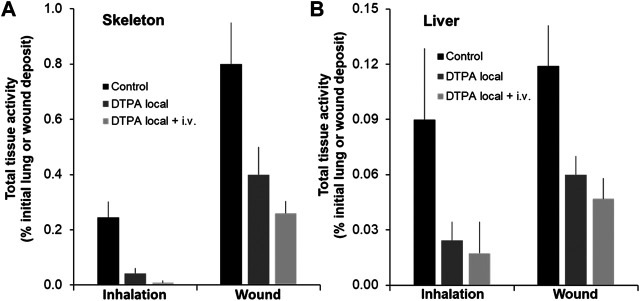
Effect of local DTPA or local DTPA followed by DTPA systemic administrations on skeletal **(A)** and liver **(B)** total activity levels following lung or wound contamination with MOX. Animals were contaminated by MOX inhalation (12 kBq) or deposition in a wound (13 kBq) as described. Local administration to lungs (DTPA powder about 5 µmol/kg) or wound (DTPA solution 30 µmol/kg) was started at 2 h (“Local”). For combined local and systemic DTPA treatment (“Local + i.v.”) animals received the local dose at 2 h then two intravenous DTPA injections per week from day one to day 20 (30 µmol/kg). Rats were euthanized 21 days following contamination. Data are expressed as a percentage of the initial total alpha activity deposit. Data are the means ± S.D. of 4–6 animals.

Following inhalation of MOX, a single local treatment to the lungs with the powder formulation of DTPA leads to an 82% reduction in the skeleton and a 73% reduction in the liver of total alpha activity (Figures 6A,B; Table 2). In the group that received the combined treatment (early local then delayed repeated systemic treatments) a further reduction in skeleton (97%; p< 0.05) was observed (Figure 6A; Table 2). Similarly following wound contamination and local DTPA treatment total activity levels were reduced by some 67 and 73% in skeleton and liver respectively (Figures 6A,B; Table 2). As observed after lung contamination the combined treatment further reduced these levels particularly in skeleton (84% p< 0.01). There was also a further inhibition in liver (81% p< 0.05) (Figures 6A,B; Table 2). It is interesting to note that after inhalation, the reduction of Pu in systemic tissues after local DTPA treatment appears greater in rats contaminated with MOX than in rats contaminated with the more soluble Pu nitrate. However, the levels of solubilized Pu/Am, and hence available for chelation, are much lower for MOX than for Pu nitrate and DTPA will be in a far higher molar excess which may explain the higher efficacy.

**TABLE 2:** Reduction of tissue activity levels by local or combined local and systemic DTPA treatment following MOX contamination.

Contaminant	DTPA treatment	Wound			Lungs		
		Leg	Skeleton	Liver	Lungs	Skeleton	Liver
MOX	Local	12 ± 15	67 ± 10	73 ± 3	6 ± 2	82 ± 6	73 ± 11
	Local + i.v.	14 ± 8	84 ± 6	81 ± 3	12 ± 3	97 ± 3	80 ± 24

Animals were contaminated by MOX inhalation or deposition in a wound and received DTPA treatment as described. Local administration (“Local”) to lungs (DTPA powder insufflation; about 5 µmol/kg) or wound (DTPA solution injection into the wound site; 30 µmol/kg) was started at 2 h. For combined local and systemic DTPA treatment (“Local + i.v.”) animals received the local dose at 2 h then two intravenous DTPA per week from day one to day 20 (30 µmol/kg). Animals were euthanized 21 days after contamination and skeletal and liver activity levels measured. Data are expressed as a percentage reduction as compared with untreated animals with four to six animals/group.

Tissue samples were also analyzed for both Pu and Am. Following MOX inhalation local DTPA treatment reduced bone Pu and Am by some 80 and 87% respectively (Table 3). The combined treatment reduced Pu and Am levels by 95 and 97% respectively (Table 3). With regard to the liver, local DTPA treatment after MOX inhalation resulted in a 61 and 78% reduction in Pu and Am (Table 3). As observed for bone, the combined treatment further reduced Pu and Am levels in the liver (81% and 92% inhibition respectively).

**TABLE 3:** Comparative efficacy of two different DTPA treatment protocols on tissue Pu and Am levels.

	Bone				Liver			
	% Initial activity	Inhibition (%)	% Initial activity	% Inhibition	% Initial activity	% Inhibition	% Initial activity	% Inhibition
Treatment	Pu		Am		Pu		Am	
(A): Inhalation								
None	0.182 ± 0.043		0.063 ± 0.016		0.041 ± 0.022		0.049 ± 0.018	
Local	0.037 ± 0.017	80	0.008 ± 0.02	87	0.016 ± 0.007	61	0.011 ± 0.004	78
Local + i.v.	0.009 ± 0.006*	95	0.002 ± 0.001*	97	0.008 ± 0.004	81	0.004 ± 0.001*	92
Difference local vs local + i.v.	*p < 0.05		* *p* < 0.05		NS		*p < 0.05	
(B): Wound								
None	0.523 ± 0.080		0.264 ± 0.075		0.052 ± 0.009		0.067 ± 0.015	
Local	0.188 ± 0.041	64	0.072 ± 0.007	73	0.024 ± 0.008	54	0.023 ± 0.009	66
Local + i.v.	0.089 ± 0.048**	83	0.039 ± 0.015***	85	0.013 ± 0.012	75	0.004 ± 0.002**	95
Difference local vs local + i.v.	**p < 0.01		*** *p* < 0.005		NS		** *p* < 0.01	

Animals were contaminated by MOX inhalation or deposition in a wound and received DTPA treatment as described. Data are expressed as a percentage of the initial total activity and as % inhibition (reduction in tissue activity as compared with untreated animals with 4–6 animals/group). Data were compared between the two treatment groups using a two-tailed unpaired t test. *p* values are also given.

Following MOX wound contamination local DTPA inhibited bone Pu and Am retention by 64 and 73% (Table 3) and observed after MOX inhalation the combined treatment further reduced bone retention of both elements (Pu 83% and Am 85% inhibition). For the liver, local DTPA injection led to a 54 and 66% reduction in Pu and Am (Table 3). The combined treatment further reduced Pu and Am levels in the liver (75 and 95% inhibition respectively).

Regardless of the contamination mode, the treatment regimen, and the organ, activity decreases in tissues were somewhat higher for Am than for Pu, presumably associated with a better chelation of Am than Pu at the primary sites of contamination as well as in the systemic compartments.

The percentages of Pu and Am in tissue samples are also compared to that of the administered MOX (27%). Similar percentages of Am were found in lungs, wound or bone whatever the route of contamination or DTPA treatment. On the contrary, an enrichment of Am in liver samples was observed similar to already shown for Urine. In this case there was a two-fold increase in untreated animals (inhalation 55 ± 5% Am; wound 56 ± 3% Am) and these values were unchanged by DTPA treatment. This was observed in other soft tissues such as kidney and testicles (data not shown). Thus in addition to the urinary excretion data, these observations are in agreement with the higher solubility of Am as compared to Pu, both from MOX or aged Pu oxides containing Am and so a higher transfer from lungs or wounds to systemic sites (Paquet et al., 2003; Ramounet-Le Gall et al., 2003; Bertelli et al., 2010; Van der Meeren and Grémy, 2010).

It is clear that either treatment regimen can reduce systemic tissue retention yet neither treatment regimen results in mobilization of measurable MOX total alpha activity from the primary site of contamination i.e., lungs or wound site (Figure 5). In the case of MOX contamination by inhalation, local DTPA will chelate the rapidly soluble transferable fraction of Pu/Am (f_r_) that represents a very small fraction of the deposited activity. The only other approach to eliminate high activity MOX particles would be to use bronchopulmonary lavage. The use of this technique as a countermeasure for lung contamination with insoluble actinides has been addressed over the years in experimental models (see Muggenburg et al., 1977; Nolibé et al., 1989; National Council on Radiological Protection and Measurements. (NCRP), 2009). A more recent paper concluded that bronchopulmonary lavage should be considered a viable treatment option for PuO_2_ intakes in order to prevent deterministic effects at lung doses over 6 Gy (Morgan et al., 2010).

With regard to wound contamination, excision of the wound site is often practiced and has indeed been employed in several cases of accidental contamination in man (Bailey et al., 2003; Laroche et al., 2010; Schadilov et al., 2010; Sugarman et al., 2018; Klumpp et al., 2020). In general surgical removal of the contaminated site is combined with both local (washing of the wound) and intravenous DTPA. The latter will prevent further retention in systemic sites resulting from contaminant blood absorption from the wound site during surgery.

An early treatment with DTPA irrespective of the actinide in all cases reduced Pu/Am levels in systemic tissues. As DTPA was given either one or 2 h after contamination, it is likely that chelation occurs in the extracellular fluid, probably at primary site of contamination and in the systemic compartment. For the more soluble actinide forms, reduction at the primary contamination site is achieved which suggests that the actinide is accessible to chelation by DTPA. In addition, DTPA was given only once reinforcing the importance of contaminant and chelator in the same biological compartments at the same time. Similar results were observed with Am nitrate in the wound model (data not shown) and indeed DTPA treatment at 30 min after contamination with either Pu or Am nitrate showed the same efficacy (data not shown). In accordance with decreased tissue levels, urinary excretion of activity was also enhanced by the different DTPA treatments. These data are in agreement with other studies either following lung or wound contamination. For MOX it appears that an additional systemic DTPA treatment further reduces tissue levels. However, tissue levels in this case are less than one percent of the administered dose as compared with 20–30% following contamination with Pu nitrate.

## Conclusion

The objective of this work was to compare different DTPA administration regimens following either pulmonary or wound contamination with different actinide forms. Firstly, the studies confirmed the differential behavior of the different forms following either pulmonary or wound contamination. The more soluble forms are absorbed to a greater extent resulting in higher urinary excretion together with greater skeleton and liver retention. Secondly, the early chelation single treatments tested show that it is possible to reduce tissue alpha-emitting actinide levels that will in consequence decrease the committed effective dose (Table 4). This is important. As a first line treatment after Pu nitrate or citrate contamination, a single, local and prompt delivery of DTPA to the primary site of contamination appears as a good alternative to intravenous administration. In addition, local treatment also reduces retention in the secondary target organs, liver and bone. Rapid administration of DTPA is thus crucial to success in order to chelate the free, transferable fraction of activity present at the primary site of contamination. Moreover, locally administered DTPA would be expected to decrease systemic retention by chelation of already transferred activity.

**TABLE 4:** Comparative efficacy of two different DTPA treatment protocols on tissue levels and urinary excretion of activity.

Contaminant	DTPA treatment	Wound			Inhalation		
		Wound site	Systemic	Urine	Lungs	Systemic	Urine
Pu nitrate	i.v.	↘	↘	↗	↘	↘	↗^1^
	Local	↘	↘	↗	↘	↘	↗^1^
MOX	Local	0	↘	↗	0	↘	↗
	Local + i.v.	0	↘↘	↗	0	↘↘	↗

“Systemic” represents skeleton plus liver the main retention organs for Pu/Am after transfer to the circulation. The arrows represent the change in percentage reduction either decrease or increase. For insoluble actinides (MOX), two arrows mean greater inhibition than one arrow (combined treatment “Local + i.v.” vs. local treatment “Local”)

^1^ Includes published data from Grémy et al., 2012.

In the case of MOX contamination, local lung or wound activity was unaffected by local or combined local and intravenous DTPA administration. Most of the activity remained at the contamination site. However, a single local DTPA administration did reduce retention in bone and liver. When combined with systemic DTPA tissue activity was further reduced. This indicates that follow-up chelation is advantageous. For the poorly soluble MOX it is clear whatever the regime used that although DTPA administration reduces systemic tissue levels it has little influence on activity levels at the primary site of contamination. In this case, radiation dose would be much greater in lungs or wound site rather than systemic organs. This leaves the question of adequate treatment for activity remaining at these primary sites of contamination, “the reservoir” unsolved. This still needs to be addressed in order to limit pathological consequences. In terms of clinical approaches and for radiation countermeasures it is therefore important to ascertain what actinide form is involved in order to administer the appropriate DTPA treatment regimen.

## Data Availability

The original contributions presented in the study are included in the article/Supplementary Material, further inquiries can be directed to the corresponding author.
